# Association Between Student Body Mass Index and Access to Sports Drinks in Minnesota Secondary Schools, 2012–2013

**DOI:** 10.5888/pcd12.150273

**Published:** 2015-11-12

**Authors:** Martha Y. Kubik, Cynthia Davey, Marilyn S. Nanney

**Affiliations:** Author Affiliations: Cynthia Davey, Marilyn S. Nanney, University of Minnesota, Minneapolis, Minnesota.

## Abstract

This ecologic study evaluated the association between school policy allowing students to purchase sports drinks from school vending machines and school stores and student body mass index (BMI). Data were from surveillance surveys of Minnesota secondary schools (n = 238) and students (n = 59,617), administered in 2012 and 2013, respectively. We used generalized linear models to assess the association between policies and mean age- and sex-adjusted BMI percentile. In adjusted multivariate analysis, school policy was positively associated with BMI percentile (*P* = .005). School policy restricting student access to sports drinks at school may contribute to decreasing consumption of sport drinks among school-aged youth and improving student weight outcomes in this population.

## Objective

Sugar-sweetened beverages (SSBs) are typically defined as some combination of regular soda, sports drinks, energy drinks, or fruit drinks. Considerable data support a link between the consumption of SSBs and weight gain and obesity (1). Policy initiatives targeting SSB availability in school settings have contributed to a decrease in consumption among school-aged youth (2,3). However, as consumption of regular soda and fruit drinks has declined, consumption of sports drinks (flavored beverages containing carbohydrates, minerals, and electrolytes intended to replace water and electrolytes lost through sweating during exercise) has grown (2–4). In the secondary school setting, sports drinks are the most commonly offered SSB (5). During 2010 and 2011, more than 80% of high school students and over half of middle school students had access to sports drinks at school (5).

Although several studies have examined the link between school policy allowing student access to SSBs at school and student weight outcomes (6), albeit with mixed results, similar studies that consider only sports drinks are lacking. The aim of this study was to evaluate the association between school policy allowing secondary school students to purchase sports drinks from school vending machines or school stores, canteens, or snack bars (hereafter called “vending machines or school stores” or VMSS) and student body mass index (BMI). This is a timely issue, because school districts nationwide are attempting to comply with the US Department of Agriculture’s Smart Snacks in School regulation, which requires all schools participating in the National School Lunch Program to offer as snacks only foods and beverages that meet specified nutrition standards (7).

## Methods

The School Obesity-Related Policy Evaluation study uses existing state and national surveillance data to examine school obesity prevention policies in Minnesota secondary schools (8). School-level data were obtained from the 2012 Minnesota School Health Profiles Principal Survey (Profiles), a biennial survey administered by the Centers for Disease Control and Prevention (CDC) and completed by middle- and high-school principals that assesses school health policies and practices (9). In 2012, 355 Minnesota schools were randomly selected to participate in the survey, and 297 schools participated. School-level demographic characteristics of participating and nonparticipating schools did not differ significantly (data not shown). Principals were asked whether students could purchase snack foods or beverages from VMSS and, if yes, whether students could purchase certain snack foods or beverages (yes or no). Options for sports drinks, soda pop and fruit drinks, chocolate candy, other candy, and non–low-fat salty snacks and baked goods (the latter 4 items combined and assessed in analyses as “snack foods”) were included.

Student-level data were obtained from the 2013 Minnesota Student Survey, administered in public schools every 3 years by the Minnesota Department of Education (10). In 2013, students in grades 5, 8, 9, and 11 participated. All 334 public school districts were invited, and 280 participated (9). District-level demographic characteristics were not significantly different between participating and nonparticipating districts (data not shown). Data from students in grades 8, 9, and 11 were used (participation rates by grade: 71%, 69%, and 62%, respectively) and included sex, age, and self-reported height and weight, which were used to calculate age- and sex-adjusted BMI percentiles, using CDC growth charts (10,11).

School-level demographic characteristics were obtained from the National Center for Educational Statistics (NCES) common core data and included percentage minority enrollment and free- or reduced-price lunch eligibility, both assessed as continuous variables. School location, categorized as city, suburb, or town or rural, was constructed using a combination of NCES and Rural–Urban Commuting Areas classification schemes (12). This study was approved by the University of Minnesota institutional review board.

The analysis was restricted to schools that completed the 2012 Profiles principals survey (n = 238) and whose students participated in the 2013 Minnesota Student Survey (n = 59,617). Consistent with an ecologic study design, school-level analyses were performed with individual student responses averaged within each school. Generalized linear models were used to assess the association between policy that allowed students to purchase sports drinks from VMSS and mean school-level BMI percentile. The model was adjusted for school-level demographics and policies allowing student purchase of soda pop/fruit drinks and snack foods. All analyses were conducted using SAS version 9.4, 2013 (SAS Institute, Inc).

## Results

Characteristics of the sample are in the Table. In adjusted multivariate analysis, a significant positive association was found between school policy allowing secondary school students to purchase sports drinks from VMSS and mean school-level BMI percentile (least squares mean = 0.59 [policy allowed sports drinks], least squares mean = 0.57 [policy did not allow sports drinks], *P* = .005).

**Table:** Characteristics of Schools (n = 238) and Students (n = 59,617), Minnesota, 2012–2013^a^

Characteristic	Value
**Schools**	
**Policy allowed purchase from VMSS**	
Sports drinks	71
Snack foods^b^	67
Soda pop/fruit drinks	45
**Mean BMI percentile (SD)**	60 (0.05)
Mean % overweight (SD) (85th to <95th percentile)	14 (4)
Mean % obese (SD) (≥95th percentile)	10 (4)
**School location**	
City	9
Suburb	17
Town or rural	74
**Mean % minority enrollment (SD)**	17 (18)
**Mean % eligible for Free or Reduced-Price Lunch Program (SD)**	34 (16)
**Students**	
**Male sex**	50
**Grade**	
8th	34
9th	36
11th	30

Abbreviations: BMI, body mass index; SD, standard deviation; VMSS, vending machines or school stores.

a Values are expressed as percentages unless otherwise indicated. Data for schools obtained from the 2012 Minnesota School Health Profiles Principal Survey; data from students obtained from the 2013 Minnesota Student Survey.

b Snack foods indicate chocolate, other candy, and non–low-fat salty snacks and baked goods.

## Discussion

Sports drinks are a recognized source of added sugars. School policy that restricts student access to sports drinks at school may contribute to decreasing consumption of sports drinks among school-aged youth and improving student weight outcomes. Recent evidence suggests that, among adolescents, the frequency of consuming sports drinks is associated with greater increases in BMI (13).

This study has several limitations. School and student surveys were cross-sectional. However, school policy data were collected 1 year before the student data, providing a reasonable time lag between policy implementation and assessment of student BMI. School-level associations may not reflect student-level associations. Other limitations were use of self-report data and a school sample that was mostly rural and white.

The Smart Snacks in School standards may contribute to a changed school food environment in which a healthy choice is the usual choice. However, a decade of school policy research suggests that the makeover will not occur overnight and that gains seen today may be lost tomorrow, especially in high schools. The popularity of sports drinks merits continued attention and further study. Empirical evidence is needed to support the advancement and institutionalization of food policy that promotes healthy food choices at school.
